# Sex-specific differences in organic anion transporting polypeptide 1a4 (Oatp1a4) functional expression at the blood–brain barrier in Sprague–Dawley rats

**DOI:** 10.1186/s12987-018-0110-9

**Published:** 2018-09-13

**Authors:** Hrvoje Brzica, Wazir Abdullahi, Bianca G. Reilly, Patrick T. Ronaldson

**Affiliations:** Department of Pharmacology, College of Medicine, University of Arizona, P.O. Box 245050, 1501 N. Campbell Avenue, Tucson, AZ 85724-5050 USA

**Keywords:** Blood–brain barrier, Drug delivery, Organic anion transporting polypeptides, Sex differences, Transporters

## Abstract

**Background:**

Targeting endogenous blood–brain barrier (BBB) transporters such as organic anion transporting polypeptide 1a4 (Oatp1a4) can facilitate drug delivery for treatment of neurological diseases. Advancement of Oatp targeting for optimization of CNS drug delivery requires characterization of sex-specific differences in BBB expression and/or activity of this transporter.

**Methods:**

In this study, we investigated sex differences in Oatp1a4 functional expression at the BBB in adult and prepubertal (i.e., 6-week-old) Sprague–Dawley rats. We also performed castration or ovariectomy surgeries to assess the role of gonadal hormones on Oatp1a4 protein expression and transport activity at the BBB. *Slco1a4* (i.e., the gene encoding Oatp1a4) mRNA expression and Oatp1a4 protein expression in brain microvessels was determined using quantitative real-time PCR and western blot analysis, respectively. Oatp transport function at the BBB was determined via in situ brain perfusion using [^3^H]taurocholate and [^3^H]atorvastatin as probe substrates. Data were expressed as mean ± SD and analyzed via one-way ANOVA followed by the post hoc Bonferroni t-test.

**Results:**

Our results showed increased brain microvascular *Slco1a4* mRNA and Oatp1a4 protein expression as well as increased brain uptake of [^3^H]taurocholate and [^3^H]atorvastatin in female rats as compared to males. Oatp1a4 expression at the BBB was enhanced in castrated male animals but was not affected by ovariectomy in female animals. In prepubertal rats, no sex-specific differences in brain microvascular Oatp1a4 expression were observed. Brain accumulation of [^3^H]taurocholate in male rats was increased following castration as compared to controls. In contrast, there was no difference in [^3^H]taurocholate brain uptake between ovariectomized and control female rats.

**Conclusions:**

These novel data confirm sex-specific differences in BBB Oatp1a4 functional expression, findings that have profound implications for treatment of CNS diseases. Studies are ongoing to fully characterize molecular pathways that regulate sex differences in Oatp1a4 expression and activity.

## Background

Effective pharmacotherapy of neurological diseases requires that drugs permeate the blood–brain barrier (BBB) and attain efficacious concentrations in the central nervous system (CNS). Indeed, current research has significantly advanced our understanding of BBB physiology such that endogenous transporters expressed at the microvascular endothelium have emerged as molecular targets that can be exploited for CNS drug delivery. Transporters that have been identified and characterized at the BBB include members of the solute carrier (SLC) and the adenosine triphosphate (ATP)-binding cassette (ABC) superfamilies of transporter proteins [1, 2]. In most cases, SLC transporters facilitate blood-to-brain transport of drugs. In contrast, ABC family members transport their substrates in the opposite direction (i.e., brain-to-blood) and, therefore, function as efflux transporters [3]. P-glycoprotein (P-gp) is a critical ABC transporter that is expressed at the BBB and is involved in limiting brain penetration of many currently marketed drugs. Many studies have attempted to improve CNS drug delivery by inhibiting transport function of P-gp; however, these strategies have not translated into the clinic due to inhibitor toxicity and poor pharmacokinetics [4–6]. Such observations suggest that direct inhibition of P-gp-mediated transport is not a viable approach for optimization of drug delivery to the brain.

The translational challenges associated with targeting P-gp indicate a need for a change in thought regarding BBB transporters. Our laboratory has proposed that a more rational strategy would be to target SLC transporters. Over the past several years, we have focused our research endeavors on characterizing regulation and functional expression of organic anion-transporting polypeptides (Oatps) [7–10], SLC family members that have several isoforms endogenously expressed at the BBB (i.e., Oatp1a4, Oatp1c1, Oatp2a1) [8, 11–16]. At the rodent BBB, the primary drug-transporting Oatp isoform is Oatp1a4 [8]. This is evidenced by studies in Oatp1a4(−/−) mice where blood-to-brain transport of clinically-relevant Oatp substrates was reduced as compared to wild-type controls [15]. Of particular significance, 3-hydroxy-3-methylglutaryl-coenzyme A (HMG-CoA) reductase inhibitors (i.e., statins) have been shown to be transport substrates for Oatp1a4 [8, 9, 17]. Statins are intriguing compounds for treatment of CNS diseases due to their neuroprotective effects [9, 18, 19]. The human orthologue of Oatp1a4 is OATP1A2, which shows similarities in localization and expression at the brain microvascular endothelium [20, 21]. Of translational significance, OATP1A2 can also transport statins such as atorvastatin and rosuvastatin [22–24].

Critical considerations in development of Oatp1a4/OATP1A2 as a molecular target for CNS drug delivery are biological variables that can modulate expression and/or activity of transporters. Specifically, sex-specific differences in Oatp functional expression must be understood in order to design effective treatment strategies where blood-to-brain drug transport is to be optimized. Indeed, such differences in SLC transporter expression and/or function have been reported in the scientific literature. For example, Cao and colleagues reported reduced expression of monocarboxylate transporter 4 (MCT4), a member of the SLC family, in liver tissue isolated from female Sprague–Dawley rats as compared to their male counterparts [25]. Additionally, this same group observed that ovariectomy caused significant differences in expression of MCT1 and MCT4, indicating a role for sex hormones in regulation of this transporter [25]. In brain tissue, expression of mRNA for organic cation transporter 3 was shown to be higher in male mice as compared to female mice [26, 27]. Several publications have reported sex-specific differences in Oatp1a4 expression in other tissues, albeit with different observations. For example, hepatic expression of Oatp1a4 was reported to be higher in female mice as compared to male mice; however, hepatic expression of this transporter was equal in male and female rats [28–30]. In contrast, Oatp1a4 mRNA and protein expression was observed to be higher in liver tissue from male Sprague–Dawley rats as compared to age-matched females [31]. Similarly, hepatic Oatp1a4 mRNA expression was 67% higher in 42-day-old male Sprague–Dawley rats [32]. This same study did not report any sex-specific differences in Oatp1a4 expression and/or activity in the kidney [32]. At present, there are not any published studies on sex-specific differences in Oatp1a4 functional expression at the BBB or on variability in Oatp-mediated CNS drug delivery between male and female experimental animals.

The goal of this study was to examine Oatp1a4 functional expression at the BBB in female Sprague–Dawley rats and in age-matched male rats. We hypothesize that biologically relevant differences in brain microvascular *Slco1a4* mRNA expression and Oatp1a4 protein expression as well as blood-to-brain transport of established Oatp substrates (i.e., taurocholate, atorvastatin) exist due to variability based on sex. Overall, data derived from this study will provide key information that will inform future work aimed at targeting Oatp-mediated transport for optimization of CNS drug delivery.

## Methods

### Materials

All chemicals utilized in our experiments were purchased from Sigma-Aldrich (St. Louis, MO) unless otherwise stated.

### Animals

Experimental animals were housed under standard 12 h light/12 h dark conditions and provided with food and water ad libitum. Animals were randomly assigned to each treatment group. Male and female Sprague–Dawley rats (200–250 g; 3 months old; Envigo, Denver, CO) were used for our studies. For experiments requiring prepubertal animals, 6-week-old male and female Sprague–Dawley rats (Envigo) were utilized.

### Ovariectomy procedure

Ovariectomy was performed on 6-week-old prepubertal female Sprague–Dawley rats using the dorsal approach as previously described [33]. Briefly, animals were anesthetized (100 mg/kg ketamine, 20 mg/kg xylazine, i.p.) and prepared for surgery. A skin incision was made on each side of an experimental animal in the lumbar region approximately 1 cm lateral to the spinal cord using a sterile surgical blade. A window in the muscular wall was made using surgical scissors and both ovaries were localized and exposed. Following ligation of the ovarian artery, ovarian vein, and uterine horn, the ovaries were removed. The remaining portion of the uterine horn was checked for bleeding and the abdominal wall was closed using 4-0 absorbable surgical thread. The skin was stapled using appropriate skin staplers. Sham operated animals (i.e., controls) underwent the same procedure but without removal of the ovaries.

### Castration procedure

Castrations were performed on 6-week-old prepubertal male Sprague–Dawley rats as previously described [33]. Briefly, animals were anesthetized with ketamine/xylazine and prepared for surgery. Testes were exposed via a transversal incision on the scrotal sac. Each testis was freed and gently excised from the scrotal sac, which enabled access to the deferent duct. The deferent duct was then ligated and both testis with their associated epididymis were removed. The remaining portion of the deferent duct was inspected for bleeding and the scrotal skin was sutured using 4-0 absorbable surgical thread. Sham operated animals (i.e., controls) underwent the same procedure but without testicular removal.

### Microvessel isolation

Microvessel isolation was performed using a protocol developed in our laboratory [10, 34]. Following euthanasia by decapitation, brains were harvested and meninges and choroid plexus were removed. Cerebral hemispheres were homogenized at 3700×*g* in 5 ml brain microvessel buffer (300 mM mannitol, 5 mM EGTA, 12 mM Tris HCl, pH 7.4) containing protease inhibitor cocktail (Sigma-Aldrich). At this time, 8 ml 26% (w/v) dextran (MW 75,000; Spectrum Chemical Manufacturing Corporation, Gardena, CA) solution was added to each homogenate sample. Samples were then thoroughly vortexed and centrifuged at 5000×*g* for 15 min at 4 °C. The supernatant was then aspirated and capillary pellets were resuspended in 5 ml of brain microvessel buffer. Dextran homogenization and centrifugation steps were repeated an additional three times to ensure appropriate quality of microvessels as demonstrated in our recent publication [10]. Following completion of dextran homogenization and centrifugation, the supernatant was aspirated and the microvessel pellet was resuspended in 5 ml of brain microvessel buffer. At this time, samples were homogenized at 3700×*g*, placed into ultracentrifuge tubes, and centrifuged at 150,000×*g* for 60 min at 4 °C. Pellets containing total cellular membranes [33] were resuspended in 500 μl of storage buffer (50% brain microvessel isolation buffer; 50% diH_2_O, v/v) containing protease inhibitor cocktail. Samples were stored at − 80 °C until further use. Purity of our microvessel preparations was shown by demonstrating enrichment in platelet endothelial cell adhesion molecule-1 (PECAM-1) as well as reduced expression of neuronal marker proteins (i.e., synaptophysin) [10, 34].

### Western blot analysis

Western blotting was performed as previously described [9, 10] with few modifications. Isolated microvessel membrane samples were quantified for total protein using Bradford reagent (Sigma-Aldrich) and heated at 37 °C for 30 min under reducing conditions (i.e., 2.5% (v/v) 2-mercaptoethanol (Sigma-Aldrich) in 1X Laemmli sample buffer (Bio-Rad, Hercules, CA) for Oatp1a4 detection. Each microvessel sample was derived from brain tissue isolated from a single experimental animal in accordance with our published protocol [34]. Following SDS-PAGE and transfer, polyvinylidene fluoride (PVDF) membranes were incubated overnight at 4 °C with primary antibodies against Oatp1a4 (anti-Oatp2, Santa Cruz Biotechnology, Dallas, TX; 1:1000 dilution) or tubulin (anti-α tubulin, Abcam, Boston, MA; 1:20,000 dilution). Microvessel purity was assessed by incubating PVDF membranes overnight at 4 °C with primary antibodies against platelet endothelial cell adhesion molecule-1 (PECAM-1; anti-CD31, Abcam, 1:100 dilution), glial fibrillary acidic protein (GFAP; anti-GFAP, Abcam; 1:20,000 dilution) or synaptophysin (anti-synaptophysin, Abcam; 1:800,000 dilution). Membranes were washed and incubated with horseradish peroxidase-conjugated anti-rabbit IgG (Jackson ImmunoResearch, 1:40,000 dilution) or anti-mouse IgG (Jackson ImmunoResearch, 1:50,000 dilution) for 60 min at room temperature. Membranes were developed using enhanced chemiluminescence (Super Signal West Pico, Thermo-Fisher). Bands were quantitated using ImageJ software (Wayne Rasband, Research Services Branch, National Institute of Mental Health, Bethesda, MD) and normalized to tubulin.

### Quantitative PCR analysis

Total RNA was extracted from brain microvessels isolated from male and female Sprague–Dawley rats using the Aurum Total RNA extraction kit (Bio-Rad, Hercules, CA). Extracted RNA was treated with amplification grade DNaseI (Bio-Rad) to remove contaminating genomic DNA. The concentration of RNA in each sample was quantified spectrophotometrically by measuring UV absorbance at 260 nm. The iScript reverse transcriptase kit (Bio-Rad) was used to synthesize first-strand cDNA. Primer pairs were prepared by Integrated DNA Technologies (Coralville, IA). Primer sequences for amplification of *Slco1a4* mRNA were as follows: forward primer 5′-GCTTCTTCATAAAAACAGCAGTAA-3′, reverse primer 5′-TGCACATGTTAATGCCAACAG-3′. Primers were designed to be complimentary to sequences located on two different exons separated by an intron to avoid amplification of genomic DNA. Quantitative PCR was performed using SYBR green master mix (Bio-Rad) on a CFX96 Touch Real-Time PCR Detection System (Bio-Rad). The quantity of the target gene (i.e., *Slco1a4*) was normalized to GAPDH using the comparative CT method (ΔΔCT). Primer sequences for amplification of *GAPDH* mRNA were as previously published by our laboratory [35].

### In situ brain perfusion

Animals were anesthetized (100 mg/kg ketamine, 20 mg/kg xylazine, i.p.) and heparinized (10,000 U/kg, i.p.). Body temperature was maintained at 37 °C using a heating pad. The common carotid arteries were cannulated with silicone tubing connected to a perfusion circuit. The perfusate was an erythrocyte-free modified mammalian Ringer’s solution consisting of 117 mM NaCl, 4.7 mM KCl, 0.8 mM MgSO_4_, 1.2 mM KH_2_PO_4_, 2.5 mM CaCl_2_, 10 mM d-glucose, 3.9% (w/v) dextran (MW 75,000), and 1.0 g/l bovine serum albumin (type IV), pH 7.4, warmed to 37 °C and continuously oxygenated with 95% O_2_/5% CO_2_. Evan’s blue dye (55 mg/l) was added to the perfusate to serve as a visual marker of BBB integrity. Perfusion pressure and flow rate were maintained at 95–105 mmHg and 3.1 ml/min, respectively. Both jugular veins were severed to allow for drainage of the perfusate. Using a slow-drive syringe pump (0.5 ml/min per hemisphere; Harvard Apparatus, Holliston, MA), [^3^H]taurocholate (1.0 μCi/ml; 10 mM total concentration; PerkinElmer, Boston, MA) or [^3^H]atorvastatin (0.5 μCi/ml; 0.013 μM total concentration; American Radiolabeled Chemicals, St. Louis, MO) was added to the inflowing perfusate. For inhibition studies, animals were perfused with erythrocyte-free modified mammalian Ringer’s solution containing transport inhibitor [i.e., 100 μM estrone-3-sulfate (E3S) or fexofenadine (FEX)] for 10 min prior to perfusion with [^3^H]taurocholate or [^3^H]atorvastatin. After perfusion, the rat was decapitated and the brain was removed. Meninges and choroid plexus were excised and cerebral hemispheres were isolated and homogenized. At this time, TS2 tissue solubilizer (1 ml) was added and each sample was allowed to solubilize for 2 days at room temperature. To eliminate chemiluminescence, 100 μl of 30% glacial acetic acid was added, along with 2 ml Optiphase SuperMix liquid scintillation cocktail (PerkinElmer, Boston, MA). Samples were measured for radioactivity on a model 1450 liquid scintillation counter (PerkinElmer).

Results were reported as picomoles of radiolabeled drug per milligram of brain tissue (C; pmol/mg tissue), which is equal to the total amount of radioisotope in the brain (C_Brain_; dpm/mg tissue) divided by the amount of radioisotope in the perfusate (C_Perfusate_; dpm/pmol): C = C_Brain_/C_Perfusate_. In a previous study using modified Ringer's solution for perfusion, the brain vascular volume in rats was shown to range between 6 and 9 μl/g brain tissue [36]. Since brain tissue was processed immediately after perfusion with radiolabeled substrate, all uptake values required correction for brain vascular volume. This was accomplished by subtracting the average vascular volume (i.e., 8 μl/g brain tissue as calculated from data reported by Takasato and colleagues) from whole-brain uptake data obtained for [^3^H]taurocholate or [^3^H]atorvastatin.

### Statistical analysis

Western blot data are reported as mean ± SD of the ratio of Oatp1a4 protein expression to tubulin protein expression where each treatment group consists of microvessel protein samples from six individual animals. These sample sizes were based on the ability to detect a 35% difference between treatment with 20% variability. Quantitative PCR data are reported as mean ± SD where each treatment group consists of microvessel mRNA samples from six individual animals. In situ brain perfusion data are reported as mean ± SD from six individual animals per treatment group. To determine statistical significance between treatment groups, Student’s *t*-test was used for unpaired experimental data. When an experiment incorporated more than two treatment groups (i.e., in situ brain perfusion inhibition studies), statistical significance was determined using one-way ANOVA followed by the post hoc multiple-comparison Bonferroni *t*-test. A value of *p* < 0.05 was accepted as statistically significant.

## Results

### Oatp1a4 mRNA and protein expression is increased in brain microvessels isolated from female adult Sprague–Dawley rats

**Fig. 1:**
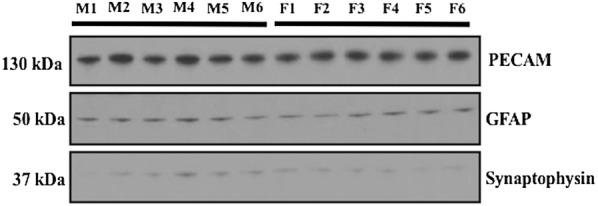
Expression of endothelial cell, astrocyte, and neuronal marker proteins in brain microvessels isolated from male and female Sprague–Dawley rats. Western blot analysis shows the presence of a single band corresponding to the endothelial marker platelet endothelial cell adhesion molecule-1 (PECAM-1; also known as CD31) (130 kDa) in male (M1–6) and female (F1–6) experimental animals. While single bands for the astrocyte marker glial fibrillary acidic protein (GFAP) (50 kDa) and the neuronal marker synaptophysin (37 kDa) in samples prepared from male and female animals, their expression was considerably lower as compared to PECAM. These observations emphasize the high purity of the microvessel preparations utilized in all subsequent experiments

**Fig. 2:**
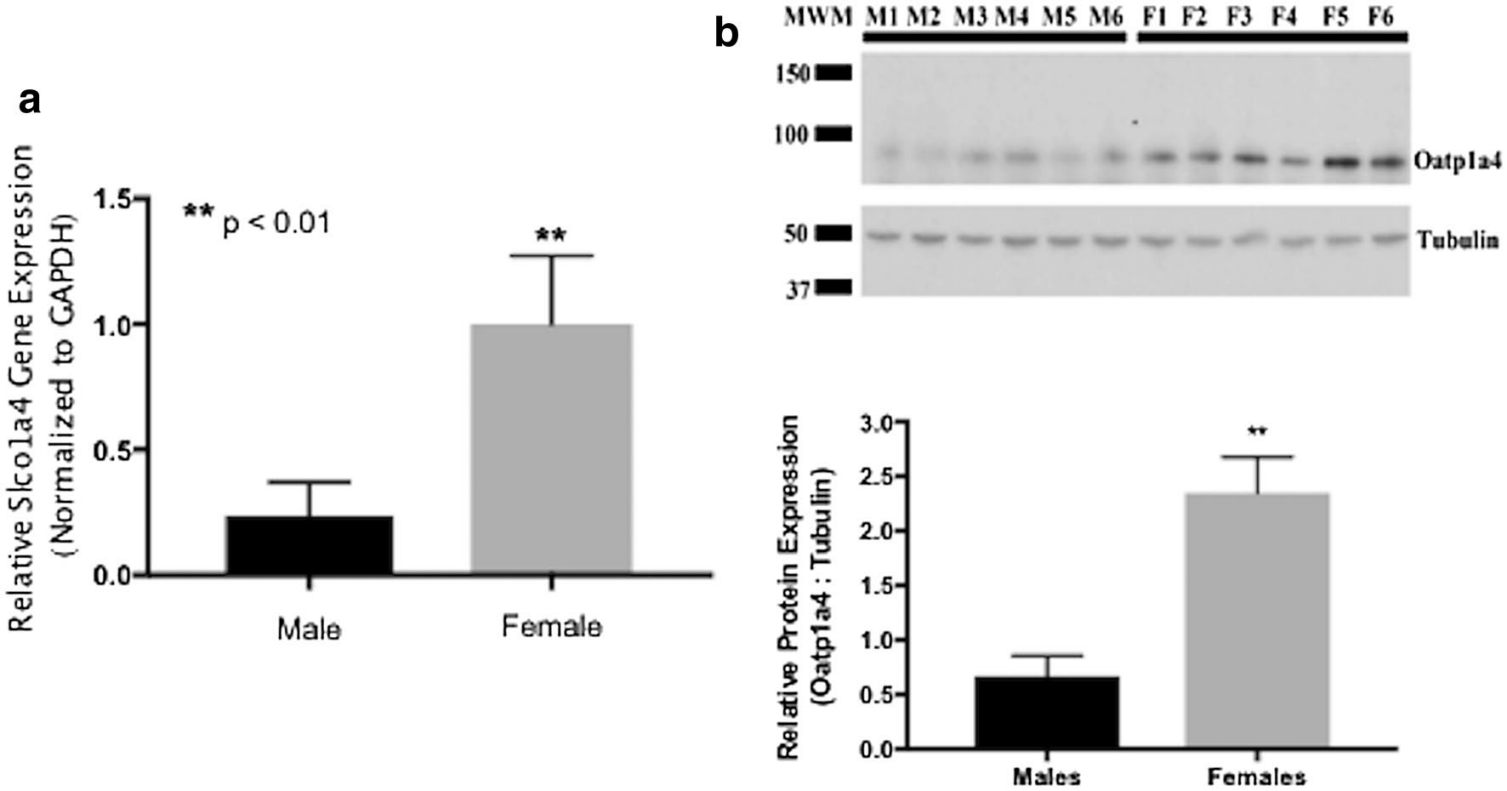
Expression of *Slco1a4* mRNA and Oatp1a4 protein in brain microvessels isolated from male and female Sprague–Dawley rats. **a** *Slco1a4* mRNA is increased in microvessels isolated from female Sprague–Dawley rats as compared to age-matched males. Results are expressed as mean ± SD of three experiments with each group consisting of mRNA samples from six individual experimental animals, **p < 0.01. **b** Western blot showed a single Oatp1a4 band at 90 kDa and a single band associated with tubulin at 50 kDa. While tubulin expression is the same between sexes, females (F1–6) show an approximately 3.5-fold higher expression of Oatp1a4 as compared to male animals (M1–6). Results are expressed as mean ± SD of the ratio of Oatp1a4 protein expression to tubulin protein expression from six animals per treatment group. *MWM* molecular weight marker. *p < 0.05

Our laboratory has previously demonstrated that functional expression of Oatp1a4 at the BBB is a critical determinant of blood-to-brain transport of drugs [7, 9]; however, it is unknown if expression and/or activity of this transporter differs between male and female experimental animals. To address this critical pharmacological question, we performed brain microvessel isolations from male and female Sprague–Dawley rats and measured expression of *Slco1a4* mRNA and Oatp1a4 protein. We have confirmed purity of our microvessel samples by demonstrating enrichment of the endothelial cell marker PECAM-1 (Fig. 1). Additionally, we show increased PECAM-1 expression in our microvessel samples as compared to expression of the astrocyte marker glial fibrillary acidic protein (GFAP) and the neuronal marker synaptophysin, which further demonstrates the purity of our microvessel preparations (Fig. 1). Microvessel sample preparations of similar purity were used for all subsequent biochemical experiments performed in this study. We observed increased expression of *Slco1a4* (4.2-fold) in rat brain microvessels isolated from female Sprague–Dawley rats as compared to their male counterparts (Fig. 2a). Similarly, Oatp1a4 protein expression was greater (3.3-fold) in microvessels isolated from female animals as compared to male Sprague–Dawley rats (Fig. 2b). The anti-Oatp2 antibody that was used in these western blot experiments recognizes an epitope corresponding to amino acids 611–660 on the Oatp1a4 protein sequence. Proper sample loading was confirmed by measurement of tubulin, a protein that polymerizes into microtubules and forms a critical component of the cytoskeleton in eukaryotic cells.

### Brain uptake of Oatp transport substrates are increased in female Sprague–Dawley rats

**Fig. 3:**
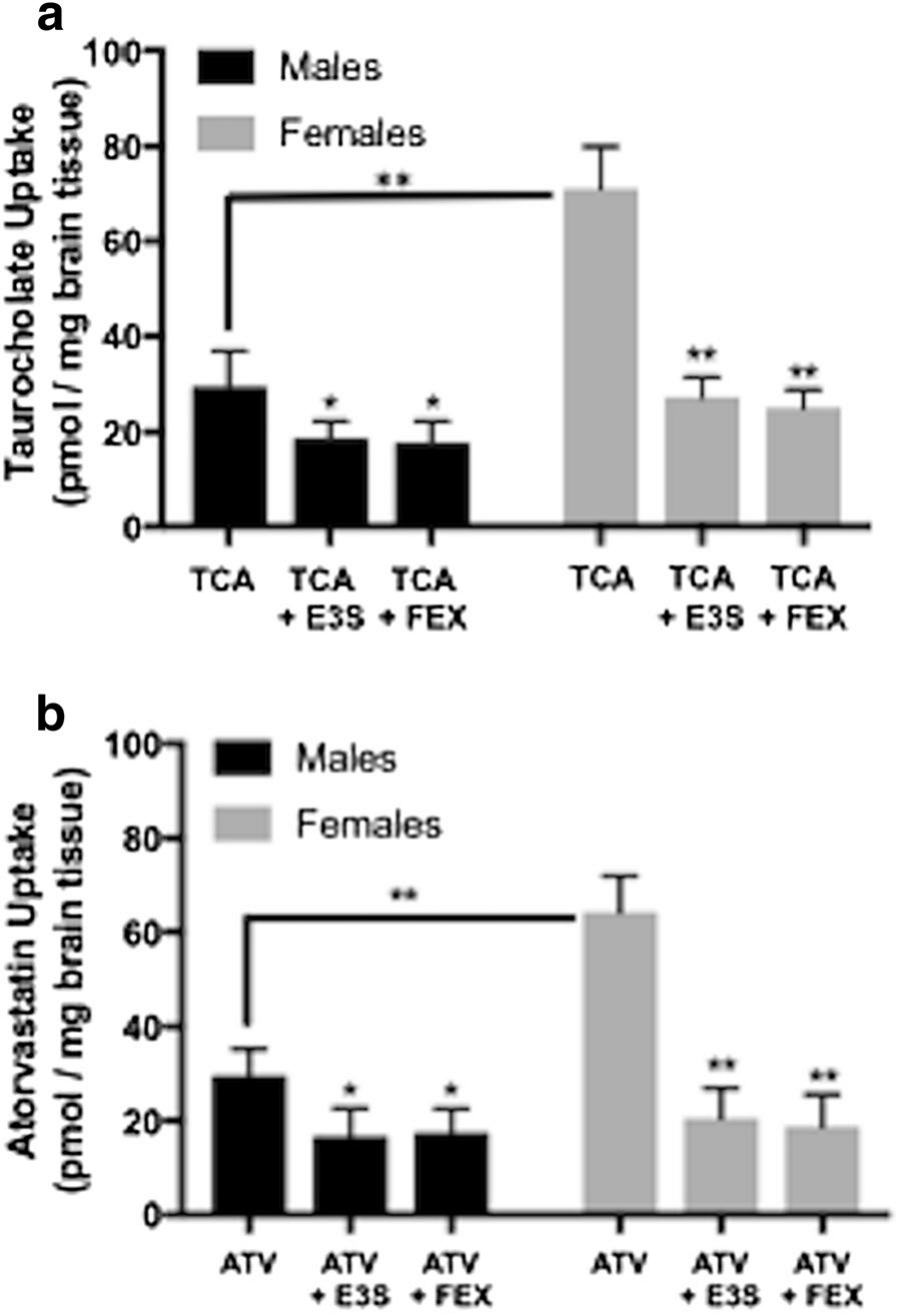
Brain uptake of the Oatp transport substrate taurocholate in male and female Sprague–Dawley rats. Uptake of [^3^H]taurocholate (TCA) or [^3^H]atorvastatin (ATV) was measured via an in situ brain perfusion study. Animals were perfused with TCA (1.0 μCi/ml) or ATV (0.5 μCi/ml) for 10 min in the presence or absence of established Oatp transport inhibitors [i.e., estrone-3-sulfate (E3S), fexofenadine (FEX)]. **a** Animals were perfused for 10 min with E3S (100 μM) or FEX (100 μM) prior to perfusion with [^3^H]taurocholate. Results are expressed as mean ± SD of six animals per treatment group. **b** Animals were perfused for 10 min with E3S (100 μM) or FEX (100 μM) prior to perfusion with [^3^H]atorvastatin. Results are expressed as mean ± SD of six animals per treatment group. For data in the absence of Oatp inhibitors, asterisks represent a statistically significant difference in Oatp substrate uptake between male and female animals. For data in the presence of Oatp inhibitors, asterisks represent data points that were significantly different from animals perfused with TCA or ATV alone. *p < 0.05; **p < 0.01

To determine whether differences in Oatp1a4 expression at the BBB between male and female animals corresponded to altered blood-to-brain transport of Oatp1a4 substrates (i.e., functional changes), the in situ brain perfusion technique was utilized. Brain uptake of [^3^H]taurocholate, a soluble bile salt and established probe drug for Oatp-mediated transport, was measured in adult male and female Sprague–Dawley rats. We have previously demonstrated that perfusion with [^3^H]taurocholate provides a unique opportunity to rigorously study differences in BBB uptake transport due to pathophysiological and pharmacological stress [7, 9, 10]. In this study, we are applying this approach to examine differences in Oatp-mediated transport due to biological variability. After a 10 min perfusion, [^3^H]taurocholate (10 μM) accumulation was significantly greater (2.2-fold) in brain tissue isolated from female Sprague–Dawley rats (Fig. 3a). To confirm that observed changes in [^3^H]taurocholate brain uptake was associated with changes in Oatp-mediated transport, male and female Sprague–Dawley rats were perfused in the presence and absence of known Oatp transport inhibitors [i.e., estrone-3-sulfate (E3S), fexofenadine (FEX)] for 10 min before perfusion with [^3^H]taurocholate. E3S significantly decreased [^3^H]taurocholate accumulation in both sexes (Fig. 3a), suggesting that blood-to-brain transport of taurocholate is an Oatp-dependent process. Similarly, we observed increased accumulation of [^3^H]atorvastatin (2.2-fold), a currently marketed Oatp transport substrate, in brain tissue extracted from female Sprague–Dawley rats as compared to their male counterparts (Fig. 3b). Both estrone-3-sulfate and fexofenadine decreased brain uptake of [^3^H]atorvastatin, an observation that indicates involvement of an Oatp-mediated process (Fig. 3b). Using high performance liquid chromatography analysis of inflow and outflow perfusate, we have previously shown that both [^3^H[taurocholate and [^3^H]atorvastatin remain intact throughout our perfusion experiments [7, 9].

### There are no differences in Oatp1a4 protein expression in brain microvessels isolated from Male and female prepubertal Sprague–Dawley rats

**Fig. 4:**
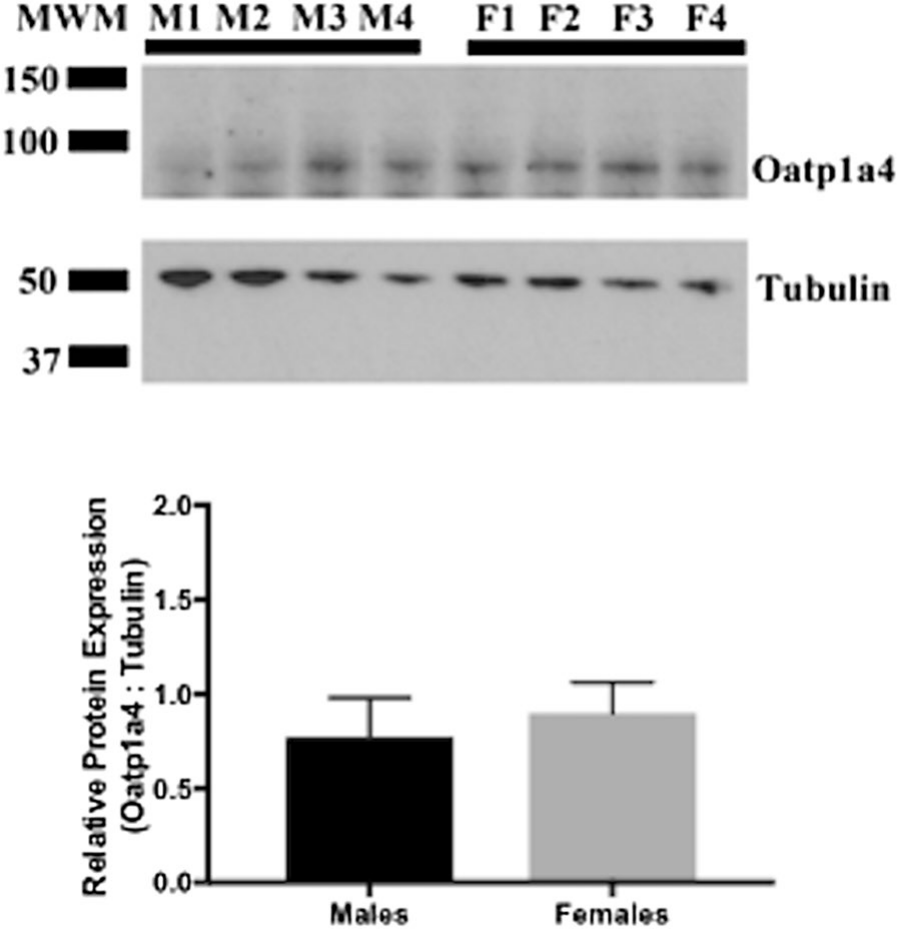
Expression of Oatp1a4 protein in brain microvessels isolated from prepubertal male and female Sprague–Dawley rats. Western blot showed a previously reported Oatp1a4 band of 90 kDa and band related to tubulin at 50 kDa in prepubertal male (M1–4) and female (F1–4) Sprague–Dawley rats. Results are expressed as mean ± SD of the ratio of Oatp1a4 protein expression to tubulin protein expression from four animals per treatment group. *MWM* molecular weight marker

Since we observed elevated Oatp1a4 protein expression in female rats as compared to male rats, we postulated that sex hormones may play a role in regulation of this critical BBB transporter. To address this hypothesis, brain microvessels were isolated from male and female prepubertal rats. These experimental animals were 6 weeks old, an age where gonadal hormones are considerably lower than observed in adult Sprague–Dawley rats [37]. We did not observe a significant difference in Oatp1a4 protein expression in brain microvessels isolated from prepubertal male Sprague–Dawley rats as compared to their female counterparts (Fig. 4). There was also no significant difference in tubulin expression in male and female prepubertal animals, which confirmed equal sample loading.

### Oatp1a4 protein expression in increased in brain microvessels isolated from castrated male Sprague–Dawley rats

**Fig. 5:**
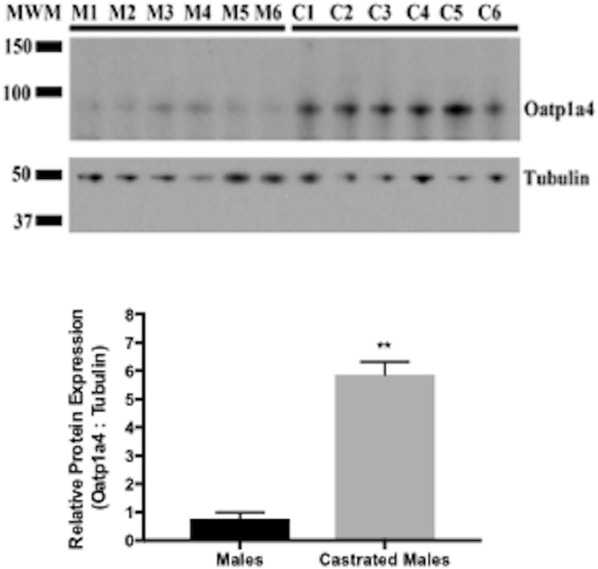
Differences in Oatp1a4 expression at the BBB in male and castrated male Sprague–Dawley rats. Western blot showed a previously reported Oatp1a4 band of 90 kDa and band related to tubulin at 50 kDa. While tubulin expression is the same between sexes, castrated males (C1–6) show an approximately 4.0-fold higher expression of Oatp1a4 as compared to control male animals (M1–6). Results are expressed as mean ± SD of the ratio of Oatp1a4 protein expression to tubulin protein expression from six animals per treatment group. *MWM* molecular weight marker. **p < 0.01

**Fig. 6:**
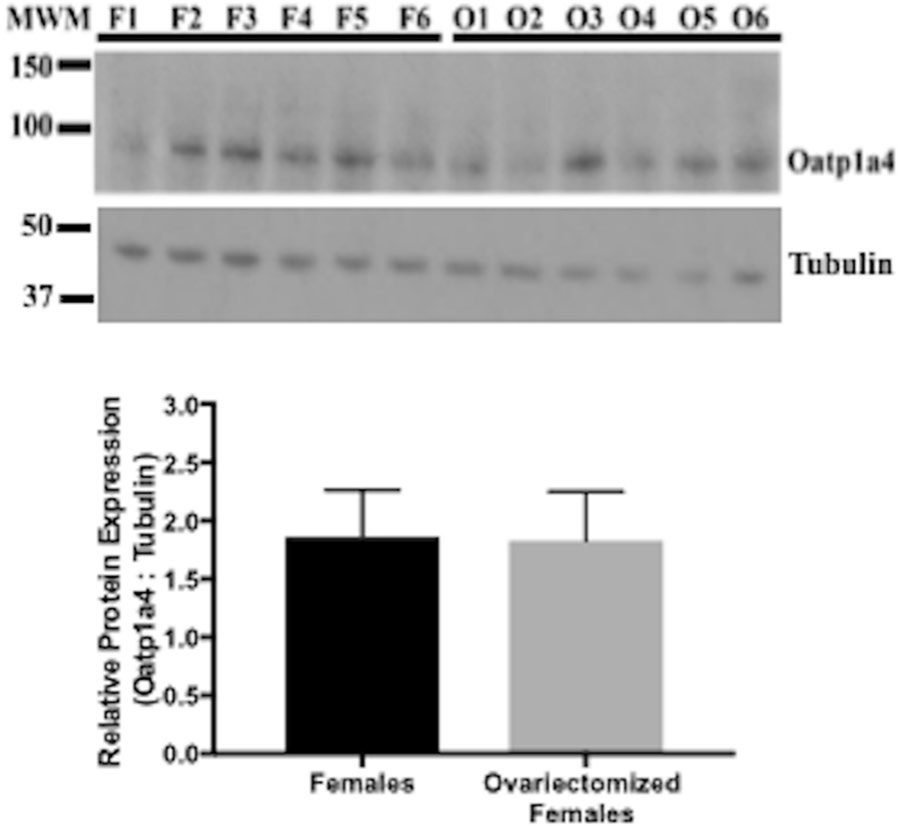
Differences in Oatp1a4 expression at the BBB in female and ovariectomized female Sprague–Dawley rats. Western blot showed a previously reported Oatp1a4 band of 90 kDa and band related to tubulin at 50 kDa. Ovariectomized females (O1–6) show no difference in expression of Oatp1a4 or tubulin as compared to control female animals (F1–6). Results are expressed as mean ± SD of the ratio of Oatp1a4 protein expression to tubulin protein expression from six individual animals per treatment group. *MWM* molecular weight marker

To further assess the role of gonadal hormones on regulation of Oatp1a4 expression at the BBB, we castrated prepubertal male Sprague–Dawley rats. These animals were permitted to recover and then were aged 8 weeks (i.e., when gonadal hormones are typically produced). At this time, castrated male rats and age-matched control animals were euthanized and brain microvessels were isolated for western blot analysis. We detected a significant increase in Oatp1a4 protein expression (up to 6.5-fold) in brain microvessels isolated from castrated males (Fig. 5). Proper sample loading was confirmed by measuring tubulin expression in microvessels isolated from castrated male Sprague–Dawley rats and from control animals. Similarly, we assessed Oatp1a4 protein expression in brain microvessels isolated from ovariectomized female Sprague–Dawley rats and age-matched controls. We observed that ovariectomy did not result in any change in microvascular Oatp1a4 protein expression (Fig. 6).

### Brain uptake of the Oatp transport substrate taurocholate is increased in castrated male Sprague–Dawley rats

**Fig. 7:**
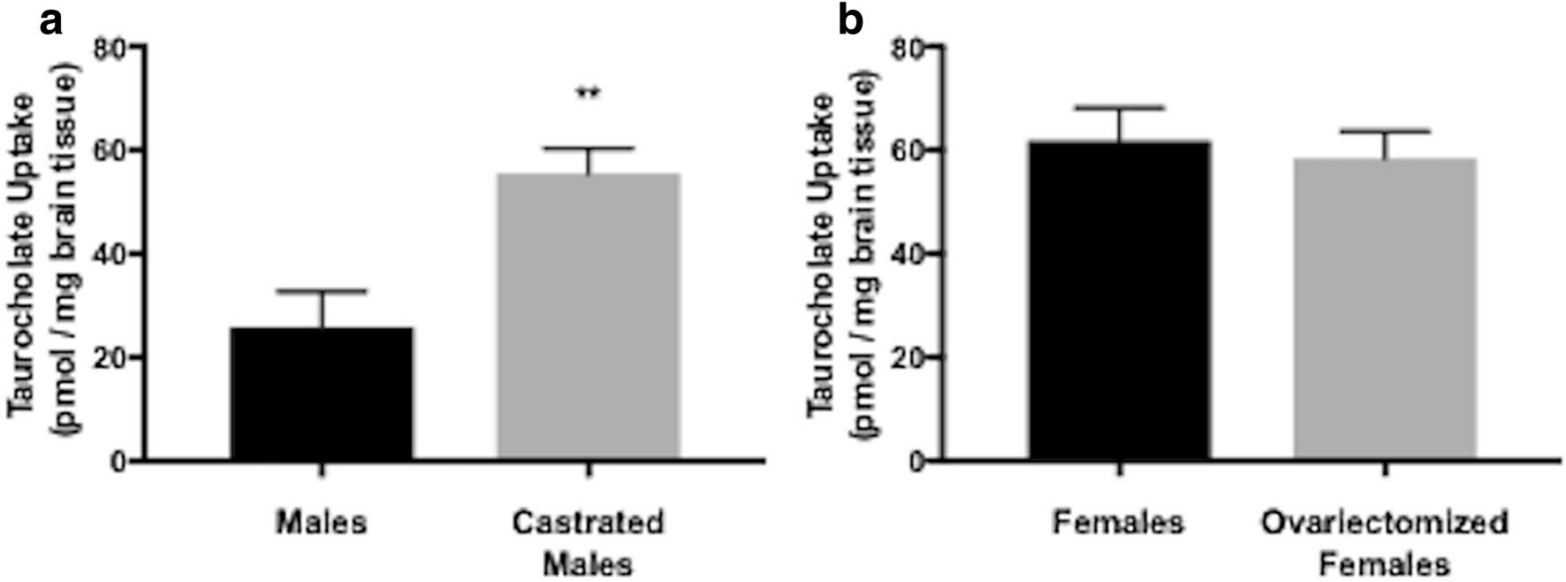
Brain uptake of the Oatp transport substrate taurocholate in castrated male and ovariectomized female Sprague–Dawley rats. Uptake of [^3^H]taurocholate was measured via an in situ brain perfusion study. Animals were perfused with [^3^H]taurocholate (1.0 μCi/ml) for 10 min. **a** Castration resulted in an increase in taurocholate uptake in castrated males compared to sham operated control males. **b** Ovariectomy of female rats did not result in significant changes in taurocholate uptake as compared to sham operated female to sham operated female rats. Results are expressed as mean ± SD from six animals per treatment group. **p < 0.01

Since we observed enhancement of brain microvascular Oatp1a4 expression in castrated male Sprague–Dawley rats, we sought to determine if this increase resulted in modification of Oatp transport activity. Therefore, we used in situ brain perfusion to measure brain uptake of the established Oatp transport substrate [^3^H]taurocholate in castrated male animals and in age-matched controls. We observed a significant increase in [^3^H]taurocholate accumulation in brain tissue isolated from castrated male Sprague–Dawley rats, suggesting an increase in Oatp transport activity (Fig. 7a). We also measured [^3^H]taurocholate uptake in brain tissue derived from ovariectomized female Sprague–Dawley rats and corresponding age-matched controls. In these animals, there was no difference in brain [^3^H]taurocholate uptake (Fig. 7b). Taken together, these data provide evidence for sex-related differences in Oatp1a4 functional expression at the BBB in Sprague–Dawley rats. Our novel results also suggest that male gonadal hormones may be a critical determinant of BBB expression and/or activity of this critical endogenous uptake transporter.

## Discussion

Sex is a significant determinant in the pathogenesis of multiple neurological diseases. This concept is best exemplified by ischemic stroke where current epidemiological data strongly indicates that stroke severity and post-stroke outcomes are highly dependent on biological variability [38]. Specifically, younger men (i.e., under the age of 45 years) are more likely to experience ischemic stroke with poorer functional recovery as compared to women of the same chronological age [39]. Incidence of stroke in women between 45 and 54 years of age increases, possibly as an effect related to changes in circulating sex hormone levels that are associated with menopause [1, 39]. From the age of 55 years onward, there are no sex differences in stroke incidence until the age of 85 years when women are at an elevated risk for ischemic stroke [39]. Within the past few years, the National Institutes of Health has emphasized the critical requirement to understand biological differences between sexes in an effort to design and develop more effective treatments for neurological diseases including ischemic stroke [40, 41]. Indeed, several studies have been reported in the scientific literature that demonstrate sex-specific differences in neuronal signaling, immune responses, and inflammation following stroke onset [41–45]. Clearly, this knowledge can inform development of new chemical entities that engage molecular targets in brain parenchyma relevant to ischemic stroke; however, such therapies cannot be rendered effective if therapeutic agents are unable to permeate the BBB and achieve efficacious concentrations in the CNS. This pharmacological fact implies a necessity to characterize sex-specific differences in BBB transport mechanisms. To date, there are no published studies examining such differences in endogenous transporter expression and/or function at the BBB. This constitutes an immense knowledge gap in the field. Indeed, understanding sex-specific differences in transporter expression offers an opportunity to provide more efficient brain delivery of neuroprotective agents to all patients afflicted by ischemic stroke.

Over the past several years, our laboratory has been interested in studying BBB transport properties of Oatp1a4, an SLC transporter that can facilitate blood-to-brain transport of multiple currently-marketed drugs [1, 7–10]. While our observations generally agreed with previous studies that reported Oatp1a4 functional expression at the BBB [14, 15, 20, 46], others showed limited, or lack of, expression of this transporter at the BBB [47, 48]. For example, Roberts and colleagues observed weak luminal Oatp1a4 staining by immunofluorescence microscopy and were unable to detect Oatp1a4 protein in microvascular protein fractions by western blotting analysis [47]. While a targeted proteomic quantitative analysis of BBB transporters in male ddy mice showed expression of Oatp1a4 at the brain microvascular endothelium [49], similar studies in rats (i.e., Sprague–Dawley and Wistar) and in non-human primates (i.e., marmosets) of both sexes did not attempt to measure Oatp1a4 expression [48]. This same group used a similar approach in human brain tissue and could not detect expression of OATP1A2 [50, 51]. Indeed, Uchida and colleagues examined brain tissue isolated from males [50] while Shawahna and colleagues studied temporal lobe tissue isolated from females [51]. It is particularly significant that the female tissue specimens examined in the Shawahna study were collected from the brain of individuals with a positive diagnosis of epilepsy. Interestingly, preclinical studies in a rodent model of chronic epilepsy showed decreased protein expression of Oatp1a4 (also known as Oatp2) in brain tissue [52]. Additionally, OATP1A2 protein expression was detected at the luminal membrane of brain microvascular endothelial cells in brain tissue isolated from male and female glioma patients [53]. The conflicting observations on OATP1A2/Oatp1a4 expression indicate a need for detailed preclinical studies to examine the role of biological variability as well as pathophysiological mechanisms on transporter localization, expression, and function at the BBB.

In the present study, we show for the first time that BBB expression of Oatp1a4 is more than threefold higher in female Sprague–Dawley rats as compared to their male counterparts. Such sex-specific differences in Oatp1a4 expression at the brain microvascular endothelium may explain differences between our data and other published studies that have utilized only male experimental animals. We also showed that sex-specific differences in Oatp1a4 protein expression at the BBB corresponds to a measurable enhancement in CNS uptake of the known Oatp transport substrates, taurocholate and atorvastatin, in female Sprague–Dawley rats. While Oatp-mediated transport has been observed in male experimental animals [14, 15, 24], ours is the first study to directly compare differences in BBB transport of two established Oatp transport substrate (i.e., taurocholate, atorvastatin) between male and female experimental animals. Using pharmacological inhibitors of Oatp-mediated transport (i.e., estrone-3-sulfate, fexofenadine), we also demonstrate that differences in taurocholate and atorvastatin brain delivery between male and female animals are primarily due to differences in Oatp-mediated transport. Sex-specific differences in atorvastatin brain uptake are particularly compelling in light of observations indicating that this highly prescribed therapeutic may act as a neuroprotectant in diseases such as Alzheimer’s disease [18] and hypoxia/reoxygenation stress [9]. Although other Oatp isoforms are expressed at the BBB, their substrate profiles do not include currently marketed drugs. Specifically, Oatp1c1 primarily transports thyroid hormones while Oatp2a1 is a prostaglandin transporter [8]. This renders our new data on Oatp1a4 particularly relevant to pharmacotherapy because it can inform translational studies aimed at improving CNS drug delivery where treatment paradigms are tailored towards an individual patient (i.e., precision medicine).

Additionally, our data demonstrates that functional expression of Oatp1a4 in male Sprague–Dawley rats is upregulated by castration, a unique observation that suggests that this BBB transporter may be repressed by testosterone. Testosterone signaling at the BBB is mediated by androgen receptors, which are well known to be expressed in brain microvascular endothelial cells [54, 55]. The classical model of androgen receptor signaling involves binding of a ligand (i.e., testosterone or its metabolite 5α-dihydrotestosterone) following permeation across the plasma membrane. Once the ligand has bound, androgen receptors undergo a conformational change that causes dissociation of heat shock proteins and translocation of the ligand–receptor complex to the nucleus. Once inside the nucleus, androgen receptor complexes dimerize, bind co-activators, and trigger transcription by binding at androgen response elements in the promoter of target genes [56]. Activated androgen receptors can also interact with caveolae and trigger kinase signaling via pathways such as Src, extracellular signal-regulated protein kinases (ERK), phosphoinositide 3-kinase (PI3K), and Akt [56]. Such non-genomic activity of androgen receptors can lead to regulation of other nuclear receptors, transcription factors, and cytoplasmic signaling events such as intracellular calcium release from the endoplasmic reticulum or mitochondria [56]. Furthermore, these non-genomic actions of androgen receptors may be critical in understanding how testosterone and its metabolites can decrease transporter functional expression at the BBB. Indeed, repression of SLC transporters in response to testosterone has been reported in other tissues. For example, organic anion transporter 3 (Oat3) expression in the kidney was shown to be elevated in male mice, upregulated following castration, and downregulated in response to testosterone treatment [57]. Similarly, renal expression of Oat2 was lower in adult male rats, increased in castrated males, and decreased following administration of exogenous testosterone [58]. While ours is the first study to provide evidence of SLC transporter repression at the BBB in response to castration, the precise mechanism as to how androgen receptor signaling regulates Oatp isoforms has not been clearly elucidated. Studies are ongoing in our laboratory to determine the complex signaling pathways involved in male sex hormone-mediated transporter repression at the BBB.

## Conclusions

In summary, our novel data show sex-specific differences in Oatp1a4 functional expression at the BBB. Our results indicate that Oatp1a4 expression and transport activity may be repressed at the brain microvascular endothelium in male Sprague–Dawley rats in response to male sex hormones (i.e., testosterone and/or its metabolites). This is the first time that sex-specific differences have been reported for an Oatp isoform at the BBB. Furthermore, these observations are particularly relevant to CNS drug delivery. Since Oatp1a4 has been shown to transport HMG-CoA reductase inhibitors (i.e., statins) such as atorvastatin, and has a similar substrate profile to its human orthologue OATP1A2 [1, 5, 8, 16], these results have considerable pharmacological implications for treatment of CNS diseases. For example, our previous work has shown that atorvastatin has potential to exert neuroprotective effects in the brain [9]; however, sex-specific differences in BBB transport may lead to variable responses to neuroprotective therapies between males and females. Therefore, it is critical to rigorously examine molecular pathways that regulate Oatps at the BBB in order to provide effective pharmacotherapy to all patients via precision medicine approaches. Indeed, studies are ongoing in our laboratory to fully understand sex differences in Oatp1a4 regulation and functional expression at the BBB, work that can inform development of novel therapeutic strategies for treatment of CNS diseases such as ischemic stroke.

## Abbreviations

ABC: ATP-binding cassette
ALK: activin receptor-like kinase
BBB: blood–brain barrier
BMP: bone morphogenetic protein
E3S: estrone-3-sulfate
ERK: extracellular signal-regulated protein kinases
FEX: fexofenadine
GFAP: glial fibrillary acidic protein
H/R: hypoxia–reoxygenation
HMG-CoA: 3-hydroxy-3-methylglutaryl-coenzyme A
MCT: monocarboxylate transporter
Oat: organic anion transporter
Oatp: organic anion transporting polypeptide
PARP: poly-ADP ribose polymerase
PECAM-1: platelet endothelial cell adhesion molecule-1
PI3K: phosphoinositide 3-kinase
P-gp: P-glycoprotein
PVDF: polyvinylidene fluoride
SLC: solute carrier
TGF: transforming growth factor
